# Effects of *crp *deletion in *Salmonella enterica *serotype Gallinarum

**DOI:** 10.1186/1751-0147-49-14

**Published:** 2007-05-08

**Authors:** Valentina Rosu, Mark S Chadfield, Antonella Santona, Jens P Christensen, Line E Thomsen, Salvatore Rubino, John E Olsen

**Affiliations:** 1 Department of Biomedical Science, University of Sassari, Viale San Pietro 43B, 07100 Sassari, Italy; 2 Department of Veterinary Pathobiology, Faculty of Life Sciences, University of Copenhagen, Stigbøjlen 4, DK-1870 Frederiksberg C, Denmark

## Abstract

**Background:**

*Salmonella enterica *serotype Gallinarum (*S*. Gallinarum) remains an important pathogen of poultry, especially in developing countries. There is a need to develop effective and safe vaccines. In the current study, the effect of *crp *deletion was investigated with respect to virulence and biochemical properties and the possible use of a deletion mutant as vaccine candidate was preliminarily tested.

**Methods:**

Mutants were constructed in *S*. Gallinarum by P22 transduction from *Salmonella *Typhimurium (*S*. Typhimurium) with deletion of the *crp *gene. The effect was characterized by measuring biochemical properties and by testing of invasion in a chicken loop model and by challenge of six-day-old chickens. Further, birds were immunized with the deleted strain and challenged with the wild type isolate.

**Results:**

The *crp *deletions caused complete attenuation of *S*. Gallinarum. This was shown by ileal loop experiments not to be due to significantly reduced invasion. Strains with such deletions may have vaccine potential, since oral inoculatoin with *S*. Gallinarum Δ*crp *completely protected against challenge with the same dose of wild type *S*. Gallinarum ten days post immunization. Interestingly, the mutations did not cause the same biochemical and growth changes to the two biotypes of *S*. Gallinarum. All biochemical effects but not virulence could be complemented by providing an intact *crp-*gene from *S*. Typhimurium on the plasmid pSD110.

**Conclusion:**

Transduction of a Tn*10 *disrupted *crp *gene from *S*. Typhimurium caused attenuation in *S*. Gallinarum and mutated strains are possible candidates for live vaccines against fowl typhoid.

## Background

The avian host specific serotype *Salmonella enterica *serotype Gallinarum consists of two biovars, *gallinarum *and *pullorum *(*S*. Gallinarum and *S*. Pullorum) [1]. They are considered the causative agents of two distinct diseases, fowl typhoid and pullorum disease, which occur especially in countries with less developed poultry industries [2]. While many western countries have succeeded with elimination of the diseases by test and slaughter, developing countries are often left with only the strategy of prophylactic treatment with antibiotics. To avoid this use of antibiotics, development of safe and effective vaccines is a priority.

The gene *crp *encodes the cAMP receptor protein (CRP), which regulates transcription of a magnitude of operons concerned with transport of sugars and catabolic functions [3]. Strains of *S*. Typhimurium and *S*. Choleraesuis with deletions in this gene are avirulent in mice [4,5] and such strains show good promises for vaccine purposes [6-8]. The importance of *crp *in the pathogenicity of the avian host specific salmonellae, and the possible protective ability of *crp *mutated strains, have never been investigated. Since deletion mutations can easily be transformed by P22 transduction, we decided to use this technique to investigate the effect of *crp *deletions in *S*. Gallinarum, taking benefit of already characterized mutations in *S*. Typhimurium.

## Methods

### Bacterial strains, plasmids and genetic manipulation

Δ*crp *mutants were constructed by generalized bacteriophage P22Ht *int *transduction from *S*. Typhimurium χ 3828 Δ*crp11- zhc1431*::Tn*10 *following standard methods [9], resulting in the strains listed in Table 1. Plasmid pSD110, which carries *S*. Typhimurium LT-2 *crp *gene including the promoter region at the 5' end [10] was used to complement Δ*crp *mutations *in trans*. The plasmid was introduced into *Salmonella *strains by electroporation as described [9]. Resistance markers were selected by using the following antibiotics concentrations: tetracycline 25 μg/ml and ampicillin 50 μg/ml.

**Table 1:** Results of mapping by PCR and analysis of expression of down stream genes by RT-PCR in *Salmonella enterica *serotype Gallinarum biovar *gallinarum *(G9, J91) and *Salmonella enterica *serotype Gallinarum biovar *pullorum *(3).

Strains	Genotype	*cpr-*PCR	Tn*10*-PCR	*argD*-PCR	*cysG-*PCR	*yfhk*- RT- PCR	*argD *RT-PCR	*cysG *RT-PCR
G9	Wt	+	-	+	+	+	+	+
G9,	Δ*crp*	-	+	-	-	-	-	-
G9	Δ*crp *+ pSD110	+	+	-	-	-	-	-
J91	Wt	+	-	+	+	+	+	+
J91	Δ*crp*	-	+	-	-	-	-	-
J91	Δ*crp *+ pSD110	+	+	-	-	-	-	-
3	Wt	+	-	+	+	+	+	+
3	Δ*crp*	-	+	-	-	-	-	-
3	Δ*crp *+ pSD110	+	+	-	-	-	-	-

(+) – PCR reactions producing amplicons of the size expected; (-) no amplicon obtained with the primers used; Wt – Wild type; (Details of primers and PCR conditions used are detailed in Table 2)

### PCR analyses and operon characterization

DNA was extracted by the FastDNA Kit (Qiagen Nordic, Ballerup, Denmark) according to the manufacturer's instructions. Δ*crp Salmonella *mutants were characterized by PCR. The presence and the sequence size of *argD, cysG *and *crp *genes in addition to the Tn*10 *insertion was investigated. Primers (DNA-technique, Aarhus, Denmark) were designed based on *Escherichia coli *and *S*. Typhimurium sequences. Amplified fragments were detected by agarose gel electrophoreses (0.8%). Primers and PCR conditions are listed in Table 2.

**Table 2:** PCR primers and conditions used to characterize *Δcrp *mutants of *Salmonella enterica *serotype Gallinarum biovar *gallinarum *(G9) and *Salmonella enterica *serotype Gallinarum biovar *pullorum *(3).

Gene	Primer	Sequence (5'→3')	Gene accession number used for primer design	PCR conditions
*crp*	crp-1	GGTGCTTGGCAAACCGC	M13773 &M13770	1
	crp-2	GCGGTTTTCGCACGTACC		
Tn*10*	crp-1	GGTGCTTGGCAAACCGC		1
	IS10as2	CGTTAAGCTGTTGAGTCG	AY583239	
*argD*	argD-1	CGGCAGAGTTTATTCCGG	AE008859	2
	argD-2	CCATACCGCGAATATCGC		
*cysG*	cysG-1	CGACTGTCTGATCGTCGG	AE008859	2
	cysG-2	CCTTTCAGGCGTACCACG		
	cysG-3	CCATGTAGAACACCAGCG		
*yhfK*	Yhfk1	CACTACGGCAAACGCTGGTG	AE008859	3
	Yhf2	AGCAGGCTGTATTTCGCTTC		
*argD*	argD-3	GAACCATGCGAACCTACATG	AE008859	3
	argD-4	TGATGAGGTGATTCTGCCTG		
*cysG*	cysG-4	AAACGCTTCTCGACTCGTGT	AE008859	3
	cysG-5	TCATAATGTCGTCGGAGACG		

1 – 94°C/5'; 94°C/30'; 60°C/1'; 72°C/2'; 72°C/10' (30 cycles)

2 – 94°C/5'; 94°C/30';58°C/1'; 72°C/2'; 72°C/10' (30 cycles)

3 – RT-PCR according to Sleator *et al*. [13].

### Sequence analysis

The sequence of *crp *in *S*. Gallinarum (G9) has recently been submitted to the GenBank database (AY594269). Alignment of the deduced protein sequences with the *S*. Typhimurium [11] and the gene product in pSD110 [10] was performed by genome blast using the NCBI web-side.

### Measurement of invasion in vivo

The invasion of mutant and wild type strains of G9 in the intestine of 10–12 week-old hens was investigated by an intestinal loop assay, as previously described [12]. Each strain was given at an average dose of 7.8 log_10 _colony forming units (CFU) and was tested in 8 different loop positions to eliminate variance due to this factor. For these assays, it was necessary to ensure that the tested bacteria were sensitive towards gentamicin. MIC values for all strains used were 0.125 μg/ml.

### Chicken infection

Groups of six-day-old chickens (Lohman Brown) with no cultural or serological evidence of *Salmonella*, using standard methods, were used for all infectivity experiments. Groups were housed individually and allowed to take feed and water *ad libitum*. The groups were infected orally with 0.5 ml of culture of *S*. Gallinarum G9 and J91 and mutants of these strains in LB broth (Difco, Brøndby, Denmark). Control birds were given LB broth without bacteria. Viable counts were made by standard culture method from inocula to determine the actual challenge dose. Inocula corresponded to approximately 7.5 log_10 _CFU. In testing for protective ability of mutated strains, birds were first given an immunizing dose of G9Δ*crp *(same dose as above) and then challenged with the same dose of wild type G9 ten days post immunization. Non-immunized birds served as the control. Chickens were observed daily and those showing signs of clinical disease were killed humanely. All experiments were conducted according to Danish legislation on animal experiments. Liver and spleen were removed from each bird and weighed. Organs were homogenized in sterile physiological saline and 10-fold dilutions were inoculated onto LB-agar with or without antibiotics. Counts were performed in duplicate. Plates were incubated at 37°C overnight (48 h for Δ*crp*) before viable counts were made.

### Phenotypic characterization

Biochemical reactions were measured using the ID32 E (bio-Merieux, Herlev, Denmark) according to the manufactures instructions. The characters selected were ornithine and lysine decarboxylase, arginine dehydrolase and acid production from manitol, sorbitol, α-galactosidase, trehalose, rhamnose, inositol, glucose, sucrose and L-arabinose. Maltose utilisation was tested on MacConkey agar (Difco) supplemented with maltose at 1% final concentration. Motility was assayed in semi-solid cysteine tryptic agar (Difco) and incubation conditions of 37°C for 18 h. H_2_S production was evaluated in triple sugar iron agar (Difco) after growth for up to 48 h at 37°C.

Growth curves were determined in LB-medium at 37°C by using both standard plate spreading and OD_520 _determination in a Bioscreen C machine (OY growth curves AB, Helsinki, Finland). Growth requirements were assayed on M63-plates with nicotinic acid (5 μg/ml) and thiamine (100 μg/ml) and with/without arginine (100 μg/ml) and cysteine (100 μg/ml).

### RT- PCR

Expression of *yhfK, argD *and *cysG *genes located downstream from *crp *were analysed by RT-PCR. RNA was extracted from strains grown in LB at 37°C to OD_450 _(0.4) using the RNeasy-kit (Qiagen Nordic). The method of Sleator *et al*. [13] was used to prepare cDNA from 1 μg RNA. For the analysis of expression, the oligonucleotides listed in Table 2 were used. To verify that the PCR band was amplified from cDNA and not contaminating chromosomal DNA in the RNA sample, a PCR reaction using the same primers was also performed using the corresponding RNA preparations as a template.

## Results and discussion

**Figure 1:**
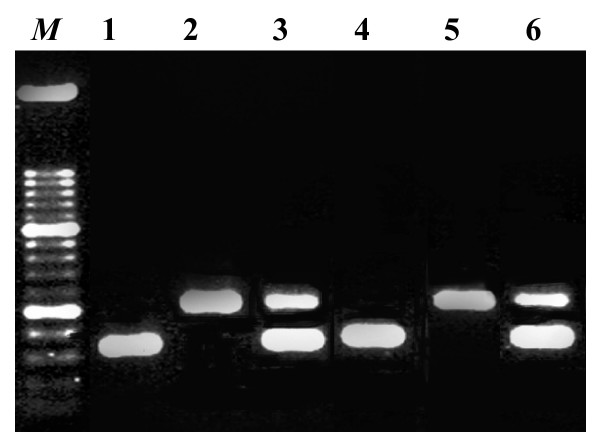
Multiplex PCR with primers crp-1, crp-2 and IS10as2. A fragment of 273 base pairs was produced inside the *crp *gene from the wild type *Salmonella enterica *serotype Gallinarum biovar *gallinarum *G9 (lanes 1 and 4) and *crp*^+ ^from pSD110 in the re-complemented strain (lane 3 and 6). A fragment of 500 base pairs was amplified between crp-1 and one of the IS sequences in Tn*10 *in the mutant (lane 2 and lane 5) and the re-complemented strain (lanes 3 and 6).

The importance of *crp *in pathogenicity of the avian host specific *S*. Gallinarum has never been investigated. Therefore the present paper aimed to study the effect of Δ*crp *in this serotype by transduction of DNA from *S*. Typhimurium deleted in this gene. To demonstrate the successful transduction, wild type, mutated and re-complemented strains of *S*. Gallinarum G9 and J91 and *S*. Pullorum 3 (Table 1) were analysed by a multiplex PCR in order to detect both the *crp *gene and the Tn*10 *insertions. A fragment of 273 base pairs was amplified inside the *crp *gene in the wild type strains, and inside the *crp-*allele on the plasmid pSD110 in the complemented strains. It further amplified a fragment of approx. 500 base pairs between the *crp *gene and one of the insertion sequences of Tn*10 *in all mutant and re-complemented strains. Results for *S*. Gallinarum G9 are shown in Figure 1. From the analysis we concluded that the wild type *crp *alleles had been exchanged with the inactivated genes in *S*. Gallinarum G9, J91 and *S*. Pullorum 3.

The sequence of *crp *in *S*. Gallinarum G9 has recently been submitted to GenBank (AY594269). Alignment of the deduced protein sequences to published sequences of *S*. Typhimurium [11] and the gene product in pSD110 [10] showed only three variable positions between the three sequences. At amino acid position 116, *S*. Gallinarum contained leucine as opposed to arginine in *S*. Typhimurium. In addition the pSD110 gene, which in the present study was used for complementation, showed deviation from the two other *crp *sequences at amino acid position 40 (leucine→serine) and position 119 (serine→alanine). Comparison of *crp *sequences across *Enterobacteriaceae*, e.g. between *S*. Typhimurium, *Shigella flexneri *and *E. coli *shows almost identical sequences [10], and in light of this, it is not surprising that *S*. Gallinarum and *S*. Typhimurium showed almost identical sequences. From this we concluded that the *crp *gene of *S*. Typhimurium very likely would be able to complement a mutated *crp *gene in *S*. Gallinarum.

Since *crp *inactivation results in attenuation in other serotypes [4,5], it was relevant to test virulence of our *crp *mutant in the specific host. The wild type *S*. Gallinarum strains used in this study has previously been shown to be virulent in chickens [14], and attenuation could therefore be attributed to the changes conferred by transduction with DNA from *S*. Typhimurium. Groups of six-day-old chickens were infected orally. The challenge experiment was repeated once with no significant difference between testings. Results of one experiment are summarised in Table 3. Birds challenged with the wild type strain of *S*. Gallinarum G9 and J91 expressed severe clinical signs of fowl cholera [2] between days four and six. They were killed humanely and pure cultures of *Salmonella *were demonstrated in liver and spleen of all birds. However, bacterial counts were not obtained; instead a value of log_10 _7 was assumed for such birds as has been generally accepted for statistical reasons in experimentation with challenge with highly virulent strains of *Salmonella*, where animals have to be sacrificed for welfare reasons [15]. This value chosen is in the area of counts usually obtained from infected birds, had they survived to day 8 [16]. The Δ*crp *mutant was attenuated and birds appeared clinically unaffected upon visual inspection throughout the 10-day observation period. All chickens that received the Δ*crp+*pSD110 strains survived the infection, and birds infected with such strains generally had bacterial counts below the detection limit in liver and spleen. Since the result was obtained twice with two different wild type strains, this shows that *crp *deletion confers attenuation to *S*. Gallinarum as has previously been reported for other serotypes [4,5].

**Table 3:** Virulence properties of *crp-*deleted mutant strain of *Salmonella enterica *serotype Gallinarum biovar *gallinarum *(G9) evaluated by presence of colony forming units (CFU) in spleen and liver following oral infection.

**Strain**	**Log_10 _CFU in spleen**	**Log_10 _CFU in liver**
G9 wild type	7.00^a^	7.00^a^
G9*Δcrp*	4.30 (± 0.63)^b^	4.06 (± 0.62)^b^
G9*Δcrp *+pSD110	1.72 (± 0.47)^c^	1.56 (± 0.31)^c^
J91 wild type	7.00 ^a^	Nd
J91*Δcrp*	4.05 (± 2.07)	Nd
J91*Δcrp *+pSD110	<2.00	Nd

^a ^– all birds in this group were sacrificed humanely due to clinical signs of disease. The log_10 _CFU was not determined and for statistical reasons these birds were given a value of log_10 _7.

a,b,c: mean CFU was statistically different by pair wise comparison between groups (p < 0.05)

ND: not done.

**Figure 2:**
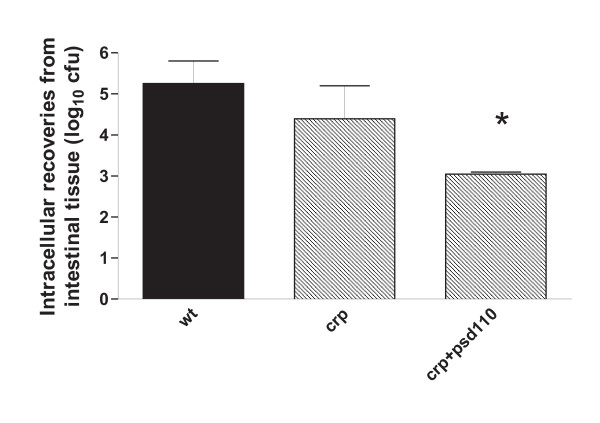
Intestinal invasion of the wild type *Salmonella enterica *serotype Gallinarum biovar *gallinarum *(G9) and Δ*crp *and Δ*crp *re-complemented with plasmid pSD110 in small intestine of hens. Experiments were replicated to allow rotation of the individual strains in different positions. Counts are expressed as log_10 _colony forming units (CFU) per biopsy of 84-mm^2 ^according to Aabo *et al*. [12]. The dose used was approximately log_10 _7.8 per loop. The invasion of the complemented strain was significantly different from the two other strains by comparison of mean CFU, as indicated by an asterix (p < 0.05). Similar results were obtained with the wild type J91 and its mutated variants.

To evaluate the role of *crp *in intestinal colonization, the invasiveness of G9 mutant strains and the corresponding pSD110 complemented strains were assessed in ligated ileal loops from hens. Figure 2 shows average counts in intestinal biopsies two hours post dosing of ileal loops with approximately 5x10E7 CFU with wild type, mutated and re-complemented strains. The invasiveness of the mutated strain was reduced compared to that of wild type, but this difference was not significant. Complementation did not restore invasiveness, and the re-complemented strain was significantly less invasive than the two other strains. The results of the invasion assay is in line with the report on *S*. Typhimurium [6] since it suggests that *crp *inactivation does not interfere significantly with the ability to invade the intestine. Contrary, *crp *mutation conferred less invasion in cell culture with a strain of *S*. Choleraesuis [4], which point to serotype differences in the way this gene influence virulence. In conclusion, it is currently unknown how *crp *inactivation confers attenuation in *S*. Gallinarum and *S*. Typhimurium. Results of the ileal loop assay suggest that the main influence is expressed at a stage beyond invasion. Yet *crp *is strongly down-regulated when *S*. Typhimurium is located inside macrophages [17].

During the challenge experiments we had observed that *S*. Gallinarum Δ*crp *mutants had reduced growth rate. This may be an important factor in the attenuation. The observation prompted us to compare the biochemical and growth changed induced in *S*. Gallinarum and *S*. Pullorum by *crp *deletion. A marked difference was observed with regard to growth rate effects between the two biovars. In *S*. Gallinarum, generation times were increased three-fold from 50 min to 180 min in the Δ*crp *mutant compared to the wild type strain. Characteristically, growth curves for the mutant strains showed lower maximum count (CFU/OD_450_) than the wild type and re-complemented strains (data not shown). pSD110 did not affect the generation time (50 min) and restored the wild type generation time in the Δ*crp *mutant (55–63 min). The complementation by pSD110 of growth rate effects observed in *S*. Gallinarum proved that *crp *from *S*. Typhimurium indeed could complement *S*. Gallinarum *in trans*. In *S*. Pullorum, on the other hand, the growth rate was not affected by Δ*crp *mutation. The generation time was 45–50 min in the wild type and 45–70 min in the mutant. The re-complemented strain showed the same generation time as the wild type. The reason for different growth rate effects between the two biovars is unknown. It may be related to the more pronounced effect of the *crp*-mutation on biochemical properties in *S*. Gallinarum compared to *S*. Pullorum (see below). The observation indicates a different role of *crp *and/or its regulatory targets for the growth of the two biovars.

Compared to the wild type strains, the Δ*crp *mutant of *S*. Gallinarum failed to utilize glucose and to decarboxylate lysine. In addition, it resulted in an inability to ferment mannose, maltose and trehalose, and to produce H_2_S in triple sugar iron agar (Table 4). The plasmid pSD110 restored the observed changes, as it has restored growth rate effects. The *S*. Pullorum wild type strain was maltose negative, which is in accordance with the reported differences between the two biovars [18]. The Δ*crp *mutant of this biovar only lost the ability to decarboxylate lysine, ferment L-arabinose and produce H_2_S in triple sugar iron agar (weak reaction in the wild type strain). In addition, the plasmid pSD110 conferred the ability to ferment trehalose and to dehydrolyse arginin in this biovar, the reason for this was not known. The phenotypic changes were complemented by providing an intact *crp *gene from *S*. Typhimurium on the plasmid pSD110.

**Table 4:** Biochemical properties of *Δcrp *strain of *Salmonella enterica *serotype Gallinarum biovar *gallinarum *(G9) and *Salmonella enterica *serotype Gallinarumbiovar *pullorum *(3).

**Strain**	**Genotype**	**Od**	**Ad**	**Man**	**So**	**Mal**	**Ga**	**Tr**	**Rh**	**In**	**Gl**	**Sa**	**Ar**	**Ld**	**H**_2_**S**	**Mot**
G9	Wt	-	-	+	-	+	-	+	-	-	+	+	+	+	+	-
G9	Wt+pSD110	-	-	+	-	+	-	+	-	-	+	+	+	+	+	-
G9	Δ*crp*	-	-	-	-	-	-	-	-	-	-	-	+	-	-	-
G9	Δ*crp*+ pSD110	-	-	+	-	+	-	+	-	-	+	-	+	+	+	-
3	Wt	+	-	+	-	-	+	-	-	-	+	-	+	+	(+)	-
3	Wt+pSD110	+	+	+	-	-	+	+	-	-	+	-	+	+	(+)	-
3	Δ*crp*	+	-	+	-	-	+	-	-	-	+	-	-	-	-	-
3	Δ*crp*+ pSD110	+	-	+	-	-	+	+	-	-	+	-	+	+	(+)	-

Od – ornithin decarboxylase; Ad – arginine dehydrolase; acid production from, Man – mannose; So – sorbitol; Mal – maltose; Ga – α-galactosidase; Tr – trehalose; Rh – rhamnose; In – inositol; Gl – glucose; Su – sucrose; Ar – L-arabinose; Ld – lysine decarboxylase; H_2_S in triple sugar iron agar; Mot – motility in semi solid agar

In order to perform at preliminary test for protective ability of mutated strains, 10 birds were first given an immunizing dose of approximately 7.5 log_10 _G9 Δ*crp *and then challenged with the same dose of wild type G9 ten days post immunization. Non-immunized birds served as control. All pre-challenged birds survived the challenge and no birds showed signs of illness, while all birds challenged with G9 without prior immunization had to be sacrificed due to severe illness. Thus the deletions caused attenuation, and oral challenge of chickens with G9 Δ*crp *completely protected against challenge with wild type G9. This finding strongly suggests that the protective ability of *crp *mutants that had been demonstrated with other serotypes and in different animal species [4-6] also holds true for *S*. Gallinarum, however, since the experiment was only conducted once, it remains to be confirmed.

Despite the successful complementation of biochemical properties of *crp *mutation, the attenuation of *S*. Gallinarum could not be complemented by *S*. Typhimurium *crp *in *trans*, and moreover, both re-complemented strains were significantly less invasive then their respective mutant strains. Given this, the current study only safely allows to conclude that transduction with the DNA fragment of *S*. Typhimurium, in which *crp *has been disrupted by Tn*10*, causes attenuation and that such strain can be good candidates for vaccines. The final proof that *cpr *is the causative gene must await disruption with site specific techniques. A similar observation has previously been reported for *S*. Choleraesuis [5]. Since the *crp *sequence of *S*. Gallinarum and *S*. Typhimurium were almost identical, and *crp *from *S*. Typhimurium complemented phenotypic changes in *S*. Gallinarum, a likely explanation is that the level of Crp is critical to the infection and expression of from a plasmid does not provide the correct level. Studies in *E. coli *have shown that different Crp mutations can prevent transcription activation at a numbers of Crp-dependent promoters and suggested that Crp can use different contacts and/or conformations during transcription at promoters with different architectures [19,20]. A less likely but possible explanation is that the three amino acid substitutions in pSD110 could influence the complementation ability to some Crp-dependent promoters, while at the same not having influence on expression from others.

Kelly et al. [4] suggested that a gene located between *argD *and *cysG*, which are located downstream from *crp *in the *S*. Typhimurium, may have been altered in some mutants in the course of the transduction, and that this could be the reason for the lack of complementation. Comparison of the gene map in *S*. Typhimurium and *S*. Gallinarum genomes shows conservation of genes and gene orders in this region (using tools available on the Web from Sanger Institute) suggesting that the most likely alteration caused by the outcome of a transduction should be only the transfer of the Tn*10 *disrupted *crp *gene. However, *S*. Typhimurium becomes auxotrophic when transduced with Δ*crp *using the same transducing fragment as in the current study [5], indicating that transduction may lead to changes in other genes that *crp*. In the current study, *S*. Gallinarum also became auxotrophic for arginine and cysteine upon transduction. In fact, all wild type strains grew on M63 minimal media with nicotinic acid and thiamine, while mutant and re-complemented strains required additional cysteine and arginin for growth (data not shown). In *S*. Typhimurium this was suggested to be due to effects on the a*rgD *and *cysG *[5]. We therefore decided to analyse the down stream region in the mutant strains of *S*. Gallinarum. PCR analysis using primers targeting *argD *and *cysG *(Table 2) amplified fragments of the expected size within the wild type strain, while no product was obtained from the Δ*crp *mutants, nor from their re-complemented strains (Table 1). This indicated that the transduced fragment had caused alterations of genes downstream from *crp*. RT-PCR was then used to analyse expression of the same genes and *yfhK*, all located downstream from *crp *in *S*. Gallinarum. Expression of these genes was only detected in wild type strains (Table 1). Thus transduction with Δ*crp *correlated with abolished expression of *argD, cysG *and *yfhK*, located immediately downstream from *crp*. Recently several *E. coli *operons, not related with catabolism, were experimentally verified as being regulated by Crp, included also the *yhfK *[21], suggesting that the lack of *yhfK *expression could be due to lack of Crp. However, we also failed to amplify the genes by ordinary PCR, suggesting that some conformational or sequence changes had happened in the region, where the primers bind.

## Conclusion

In conclusion transduction of a *crp *deletion from *S*. Typhimurium to *S*. Gallinarum by P22 transduction caused attenuation and the mutated strain may have vaccine potentials, since orally infected chickens survived challenge with wild type strains.

## Abbreviations

The following abbreviations were have been used for *Salmonella *serotypes and biovars:

*S. enterica *serotype Typhimurium: *S*. Typhimurium

*S. enterica *serotype Choleraesuis: *S*. Choleraesuis

*S. enterica *serotype Gallinarum biovar *gallinarum*: *S*. Gallinarum

*S. enterica *serotype Gallinarum biovar *pullorum: S*. Pullorum

## Competing interests

The author(s) declare that they have no competing interests.

## Authors' contributions

VR and AS performed mutations and characterized strains biochemically and by PCR. They performed virulence characterization in collaboration with MSC and JPC. LET performed RT-PCR. SR and JEO contributed significantly to the design of the study and JEO drafted the manuscript. All authors contributed to the wording of the final version of this manuscript.
